# Piezoelectric MEMS Acoustic Transducer with Electrically-Tunable Resonant Frequency

**DOI:** 10.3390/mi13010096

**Published:** 2022-01-08

**Authors:** Alessandro Nastro, Marco Ferrari, Libor Rufer, Skandar Basrour, Vittorio Ferrari

**Affiliations:** 1 Department of Information Engineering, University of Brescia, 25123 Brescia, Italy; marco.ferrari@unibs.it (M.F.); vittorio.ferrari@unibs.it (V.F.); 2 CNRS, Grenoble INP, TIMA, University Grenoble Alpes, 38000 Grenoble, France; libor.rufer@univ-grenoble-alpes.fr (L.R.); skandar.basrour@univ-grenoble-alpes.fr (S.B.)

**Keywords:** MEMS, piezoelectric, PiezoMUMPs, acoustic transducer, tunable, resonant frequency, finite element modelling

## Abstract

The paper presents a technique to obtain an electrically-tunable matching between the series and parallel resonant frequencies of a piezoelectric MEMS acoustic transducer to increase the effectiveness of acoustic emission/detection in voltage-mode driving and sensing. The piezoelectric MEMS transducer has been fabricated using the PiezoMUMPs technology, and it operates in a plate flexural mode exploiting a 6 mm × 6 mm doped silicon diaphragm with an aluminum nitride (AlN) piezoelectric layer deposited on top. The piezoelectric layer can be actuated by means of electrodes placed at the edges of the diaphragm above the AlN film. By applying an adjustable bias voltage *V*_b_ between two properly-connected electrodes and the doped silicon, the d31 mode in the AlN film has been exploited to electrically induce a planar static compressive or tensile stress in the diaphragm, depending on the sign of *V*_b_, thus shifting its resonant frequency. The working principle has been first validated through an eigenfrequency analysis with an electrically induced prestress by means of 3D finite element modelling in COMSOL Multiphysics^®^. The first flexural mode of the unstressed diaphragm results at around 5.1 kHz. Then, the piezoelectric MEMS transducer has been experimentally tested in both receiver and transmitter modes. Experimental results have shown that the resonance can be electrically tuned in the range *V*_b_ = ±8 V with estimated tuning sensitivities of 8.7 ± 0.5 Hz/V and 7.8 ± 0.9 Hz/V in transmitter and receiver modes, respectively. A matching of the series and parallel resonant frequencies has been experimentally demonstrated in voltage-mode driving and sensing by applying *V*_b_ = 0 in transmission and *V*_b_ = −1.9 V in receiving, respectively, thereby obtaining the optimal acoustic emission and detection effectiveness at the same operating frequency.

## 1. Introduction

Acoustic transducers based on micro electro-mechanical systems (MEMS) represent a lively research field and, at the same time, provide a significant number of concrete solutions and commercial devices. Specifically, thanks to the advantages provided by MEMS technology such as compact sizes, low production costs and high compatibility with IC technology [1], acoustic MEMS transducers have been extensively employed in different applications. In biomedical fields they have been exploited to monitor heart and lungs sounds [2,3] and for cochlear implants [4]. In the livestock sector MEMS acoustic transducers have been used to estimate the state of health of animals [5] while in industrial fields they have been used for noise and vibration measurements [6], as resonant photoacoustic combustion gas monitors [7] or as hydrophones for pipeline leak detection [8]. In recent years MEMS acoustic sensors and actuators have been extensively produced for consumer applications as microphones for wearable devices [9,10] and voice controllable systems [11,12] or as microspeakers for in-ear devices [13,14]. MEMS acoustic transducers rely on the conversion of energy between mechanical/acoustic and electrical domains which can be achieved by different transduction mechanisms. The most commonly used are the electrostatic [15,16,17], piezoresistive [18] and piezoelectric [19,20,21,22,23] mechanisms. Specifically, the piezoelectric transduction mechanism compared to other principles has higher energy density and does not require polarization voltages [24]. A crucial parameter for the development of an acoustic piezoelectric MEMS transducer is the resonant frequency of the MEMS structure, since it influences the frequency response of the device [25] and also represents the condition around which the transducer has the maximum transmission and receiving effectiveness in narrowband operation. Usually, a piezoelectric transducer near resonance can be described by an equivalent electrical circuit composed of two parallel branches, i.e., the motional and the electrical branches [26]. The mechanical properties such as the effective mass, mechanical damping, and stiffness of the resonator are modelled by the motional branch, while the electrical branch is associated to the capacitance arising from the dielectric nature of the piezoelectric material [27]. Therefore, the frequency response of the device is characterized by two resonances which differ in value named the series resonant frequency *f*_s_ and parallel resonant frequency *f*_p_ [28]. According to such an equivalent circuit, a piezoelectric acoustic transducer, under voltage excitation, exhibits the highest transmitting output at the series resonance, while, under voltage readout, it displays the highest receiving sensitivity at the parallel resonance, shifted to a higher frequency [29]. However, when only a single piezoelectric element is used as a transceiver for both transmitting and receiving acoustic signals at resonance, a dynamic frequency tuning would be desirable to obtain the maximum transmitting-receiving effectiveness [30]. Typically, to reach that goal, additional electrical tuning circuits or matching networks can be dynamically added to the transducer [31,32,33,34]. However, in systems with several transducers such as in arrays, multiple networks are required to connect all transducer elements thus increasing the complexity of the overall system. Other solutions rely on the application of DC voltages to change mechanical properties of the transducer allowing to increase the bandwidth merging two closely-spaced resonance modes [35].

In this context, the present work proposes a technique to obtain an electrically-tunable matching between the series and parallel resonant frequencies of a single piezoelectric MEMS acoustic transducer to increase the effectiveness of acoustic emission/detection in voltage-mode driving and sensing. A DC bias voltage is applied to the piezoelectric layer inducing a controllable stress thus leading to a matching of the series and parallel resonant frequencies in transmitter and receiver modes. At first, the working principle has been validated through a 3D finite element modelling employing a parametric two-step study to compute the first flexural mode of the structure considering the influence of a static electric field applied across the piezoelectric layer. Then, the proposed technique has been experimentally verified by configuring the fabricated MEMS acoustic transducer in both emitter and receiver modes with applied DC bias. Finally, the matching of the acoustic emission and detection characteristics with the same operating frequency in voltage-mode driving and sensing has been experimentally achieved.

The paper is organized as follows: fabrication technology and device design (Section 2), finite element analysis of the piezoelectric MEMS device (Section 3), experimental results (Section 4) and conclusions (Section 5).

## 2. Fabrication Technology and Device Design

**Figure 1:**
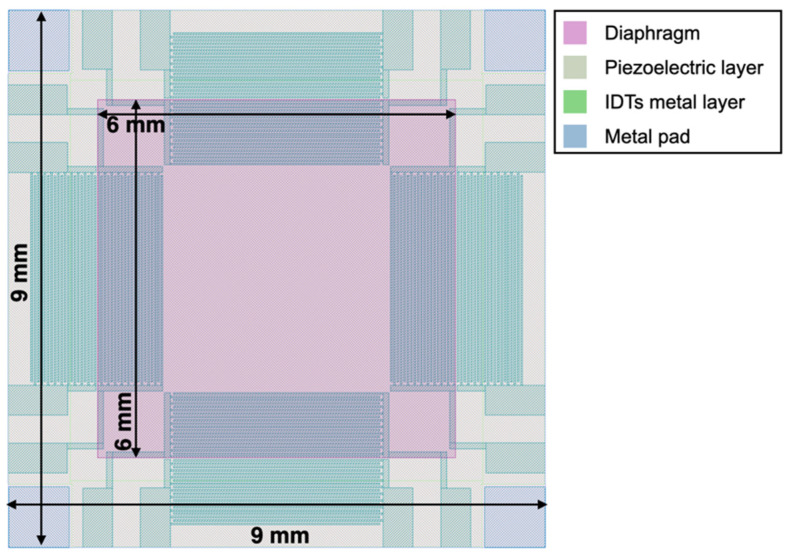
Top view image of the proposed piezoelectric micro electro-mechanical systems (MEMS) taken from the graphic design system (GDS) file.

The top view of the proposed piezoelectric MEMS device, taken from the graphic design system (GDS) file, is reported in Figure 1. The proposed 9 × 9 mm MEMS device exploits a 6 × 6 mm highly doped silicon diaphragm with an aluminum nitride (AlN) piezoelectric layer deposited on top that can be actuated by eight interdigital transducers (IDTs), each composed by two interlocking metal comb-shaped arrays of twenty equally spaced fingers. The IDTs are placed on the inner and outer edges of the diaphragm and disposed symmetrically with respect to its centre. The doped silicon layer can be electrically connected employing the four metal pads located at the die corners. The layout of the device, and specifically of the IDTs, has been designed to create a general-purpose piezoelectric MEMS platform exploitable in different applications. In particular, the IDTs have been exploited to generate Lamb waves in the diaphragm at frequencies in the megahertz range to drive mechanical vortexes in liquids for biological applications [36].

In this work, on the other hand, to induce a planar static compressive or tensile stress in the diaphragm and to excite/detect acoustic signals, the IDTs have been employed as top plates over the piezoelectric layer, while the highly doped silicon layer has been used as a common bottom plate, thus configuring the electrodes to produce in all respects parallel-plate transducers. Therefore, the IDTs layout, and specifically the spacing between two consecutively electrodes, does not affect the presently proposed application.

**Figure 2:**
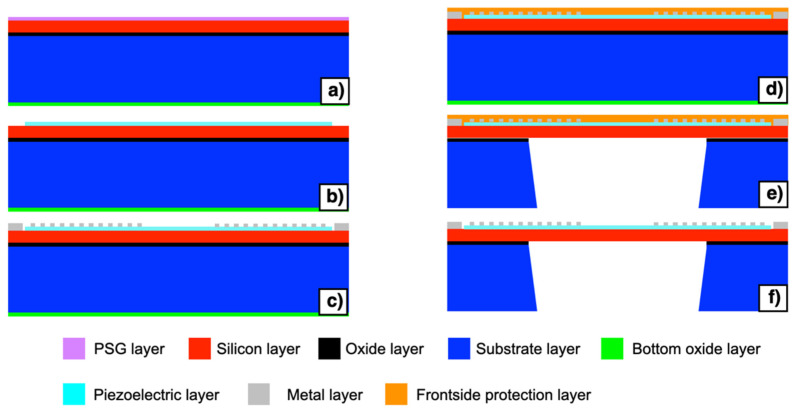
Manufacturing process steps (**a**–**f**) of the piezoelectric multi-user MEMS processes (PiezoMUMPs) technology involved for the fabrication of the piezoelectric MEMS device.

The piezoelectric MEMS has been manufactured by employing the piezoelectric multi-user MEMS processes (PiezoMUMPs) technology developed by the MEMSCAP foundry [37]. The manufacturing process steps are illustrated in Figure 2a–f. The process employs a 150 mm <100> oriented silicon-on-insulator (SOI) wafer where the silicon, the oxide and the silicon substrate have thicknesses of 10 ± 1 μm, 1 ± 0.05 μm and 400 ± 5 μm, and are shown in Figure 2 with red, black and blue colours, respectively. A bottom side oxide layer, shown in green colour, is also present on the starting substrate. The process begins with the doping step of the wafer reported in Figure 2a. This step involves the deposition of a phosphosilicate glass (PSG) layer, shown in purple colour, and its annealing at 1050 °C for 1 h in argon. The PSG layer is subsequently removed using wet chemical etching. The piezoelectric film lift-off occurs as the second step of the manufacturing process, reported in Figure 2b. The piezoelectric film consisting of 0.5 μm of aluminum nitride (AlN), shown in cyan colour, is deposited over the wafer by reactive sputtering. The third step involves the pad metal lift-off, reported in Figure 2c. A metal stack consisting of 20 nm of chrome (Cr) and 1000 nm of aluminum (Al), shown in grey colour, is deposited by beam evaporation.

**Figure 3:**
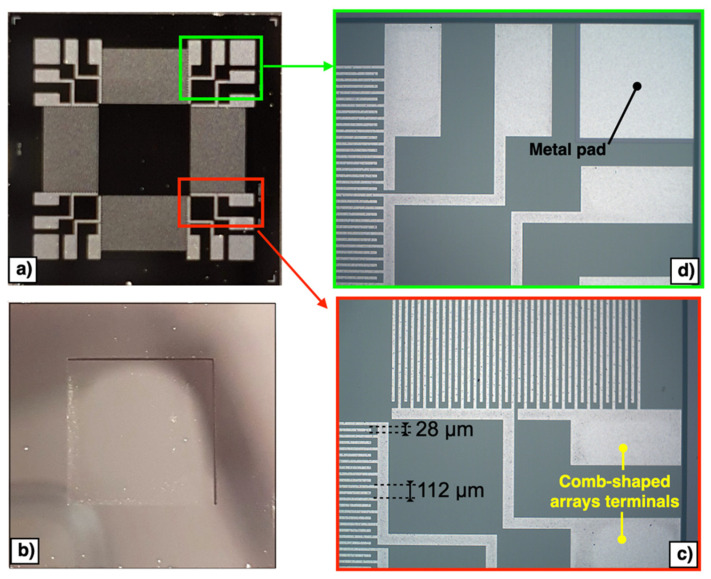
Top (**a**) and bottom (**b**) views of the fabricated piezoelectric MEMS device. Enlarged images of the comb-shaped arrays terminals (**c**) and metal pad (**d**).

A front side polyamide protection coat, shown in Figure 2d in orange colour, is then applied to the top surface of the wafer. The wafer is then reversed, and the substrate layer is lithographically patterned from the bottom side, as reported in Figure 2e. Reactive ion etching (RIE) is used to remove the bottom side oxide layer while a DeepRIE (DRIE) is subsequently used to etch the substrate layer up to the silicon layer. Finally, the front side protection material is stripped during the release step, as reported in Figure 2f. Top and bottom views of the fabricated piezoelectric MEMS device are reported in Figure 3a,b, respectively. The proposed device embeds electrical terminals for each comb-shaped arrays of fingers, while four electrically shorted metal pads are placed in each corner of the device to contact the highly doped silicon layer beneath the AlN piezoelectric layer, as reported in Figure 3c,d, respectively. Each comb-shaped array of the IDTs includes fingers with width of 28 μm and pitch of 112 μm, as reported in Figure 3c.

**Figure 4:**
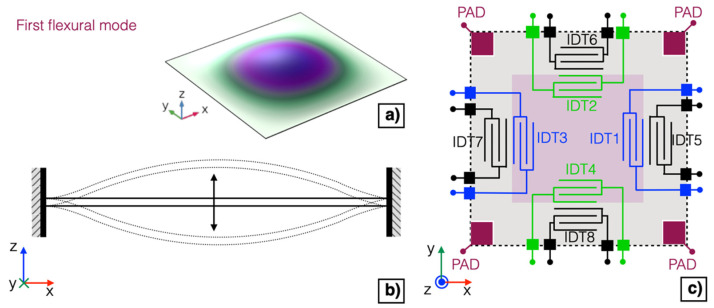
Displacement (**a**) and section view (**b**) of a fully-clamped square plate vibrating at the first flexural mode. Simplified schematic of the proposed piezoelectric MEMS device (**c**).

The possibility to obtain an adjustable matching between the series and parallel resonant frequencies of the first flexural mode of the piezoelectric MEMS transducer to increase the effectiveness of acoustic emission/detection has been investigated by electrically tuning the mechanical characteristics of the diaphragm. The displacement and the section view of a fully-clamped square plate vibrating at the first flexural mode is reported in Figure 4a,b, respectively.

Two IDTs, namely IDT1 and IDT3, placed on the opposite inner edges of the diaphragm, shown in the simplified schematic of Figure 4c in blue colour, have been shorted and employed as a top plate over the piezoelectric layer.

The doped silicon layer, contactable by employing the metal pads shown in Figure 4c in dark red colour, has been grounded and employed as a bottom plate. The four IDTs on the outer edges of the diaphragm, i.e., IDT5–IDT8, shown in black colour, have not been employed and thus left unconnected. The remaining two IDTs, namely IDT2 and IDT4, on the inner edges of the diaphragm, shown in green colour, have been adopted for excitation or detection of acoustic signals.

**Figure 5:**
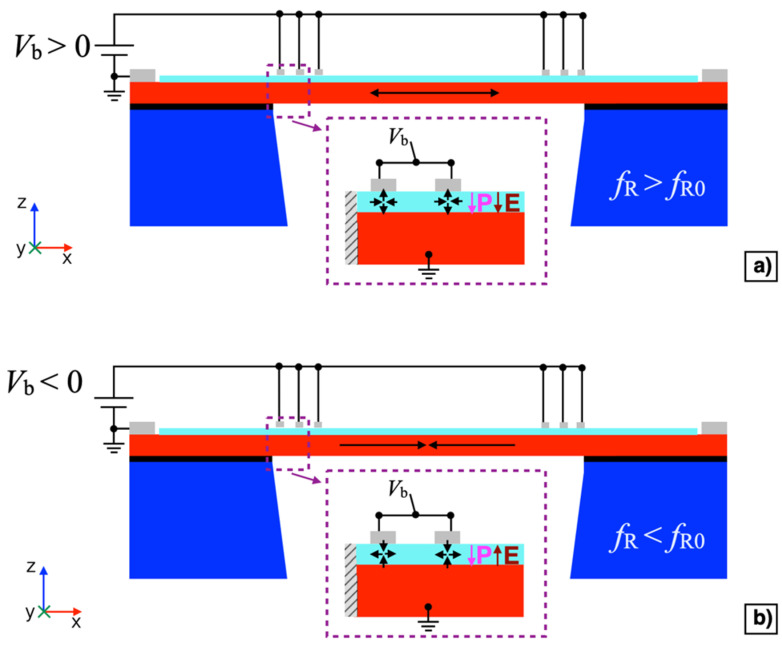
Cross-sectional view of the piezoelectric MEMS device with positive (*V*_b_ > 0 V) (**a**) and negative (*V*_b_ < 0 V) (**b**) bias voltage.

By applying a DC bias voltage *V*_b_ between IDT1 shorted with IDT3 and the silicon pad, it is possible to exploit the d31 mode in the AlN film to induce a planar static compressive or tensile stress in the diaphragm, depending on the sign of the bias voltage *V*_b_, as reported in Figure 5a,b, respectively. The application of an electrically controllable mechanical stress allows the mechanical resonant frequency *f*_R0_ of the diaphragm to be shifted thus leading to tunable resonant frequency. Studies have proven that a stress induced to a clamped square plate leads to variations of the frequencies of its vibrational modes, including the first flexural mode [38,39]. Considering the spacing of 28 μm between two consecutive fingers and the thickness of the AlN layer of 0.5 μm, the electric field **E** induced within the AlN layer below each finger can be assumed as in the configuration of parallel plates, where the top plate is the corresponding finger, and the bottom plate is the highly doped silicon layer. Given the piezoelectric polarization vector **P** oriented along the negative direction of the z-axis, by applying a positive bias voltage *V*_b_ > 0, an expansion along the z-axis and a contraction along the x-axis of the piezoelectric material is produced at each finger, as reported in the inset of Figure 5a.

Consequently, a planar static tensile stress is induced into the diaphragm as indicated by the arrows, thus increasing the mechanical resonant frequency *f*_R_, i.e., *f*_R_ *> f*_R0_. On the contrary, by applying a negative bias voltage *V*_b_ < 0 a contraction along the z-axis and an expansion along the x-axis of the piezoelectric material is produced at each finger, as reported in the inset of Figure 5b. Consequently, a compressive stress will be induced into the diaphragm as indicated by the arrows, thus decreasing the mechanical resonant frequency *f*_R_, i.e., *f*_R_ < *f*_R0_.

## 3. Finite Element Analysis of the Piezoelectric MEMS Device

**Figure 6:**
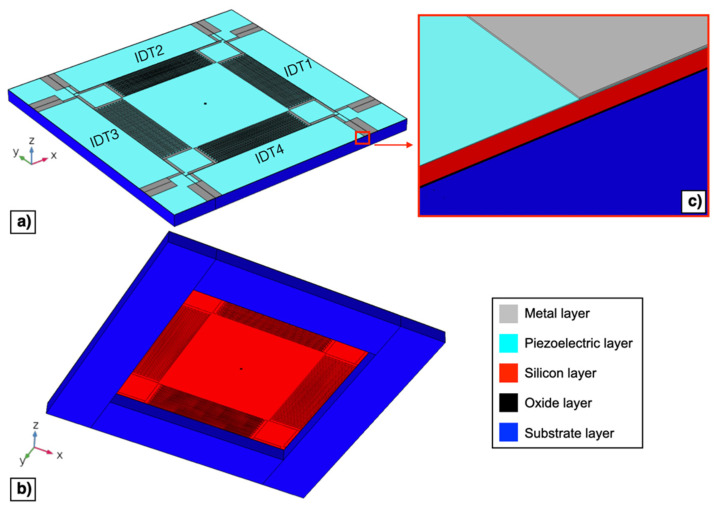
Top (**a**) and bottom (**b**) 3D model views of the proposed piezoelectric MEMS device. Enlarged image of the layers employed in the simulation model (**c**).

The electro-mechanical behaviour of the piezoelectric MEMS device described in Section 2 has been investigated by means of 3D finite element modelling in COMSOL Multiphysics^®^. Top and bottom views of the developed 3D model of the device are reported in Figure 6a,b, respectively.

Figure 6c reports an enlarged view of the structural layers that have been included in the 3D model. The nominal dimensions reported in Section 2 have been considered, i.e., neglecting tolerances in layer thicknesses produced by the manufacturing process. In the reported 3D model, the metal layer has been considered as made by Al, thus Cr has been neglected, since the Al thickness is 50 times higher than the Cr thickness. The four IDTs on the outer edges of the diaphragm have not been included in the model since, as described in Section 2, they have not been actuated and they do not affect the mechanical properties of the diaphragm to any significant extent. The SiO_2_ has been used as the oxide layer material while Si <100> has been adopted for the substrate and the silicon layer.

The piezoelectric coefficients d_31_ = −2.78 pC/N and d_33_ = 6.5 pC/N have been specified for the AlN piezoelectric layer as reported in [22,35]. A rotation of 180 deg around the x-axis of the coordinate system has been adopted for the piezoelectric layer to correctly align the poling direction with the negative direction of the z-axis. The piezoelectric effect has been considered by including in the simulation the piezoelectric multiphysics which combines the solid mechanics with the electrostatics physics.

Regarding the solid mechanic physics, a fixed boundary constraint has been applied to the bottom surface of the substrate while for the piezoelectric layer a strain-charge constitutive relation has been specified including the AlN material properties. The gravity constraint has been applied to the domains of the whole structure.

**Figure 7:**
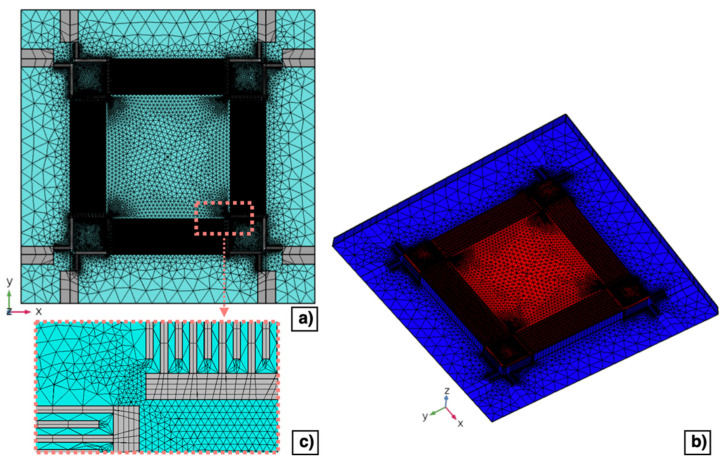
Top (**a**) and bottom (**b**) view of the mesh domain developed for the 3D model of the piezoelectric MEMS transducer. Enlarged view of the mesh of the comb-shaped arrays (**c**).

Regarding the electrostatics physics, a charge conservation boundary condition has been applied to the AlN layer. Terminal constraints have been specified to the domain of each comb-shaped arrays of fingers. The metal pads placed in the device corners to contact the silicon layer beneath the piezoelectric layer have not been included in the 3D model since a ground constraint has been applied to the top surface of the silicon. The mesh domain has been carefully designed to obtain a convergent solution while reducing the computational workload. Top and bottom views of the mesh domain are shown in Figure 7a,b, respectively. Layers that compose the diaphragm have been studied with a finer mesh, while layers laid on the outer edges of the diaphragm with a coarser mesh, as reported in Figure 7c. Specifically, top surfaces of the metal and AlN layers that compose the diaphragm have been meshed with a mapped resolution distribution of 1 μm and with a free triangular minimum element size of 36 μm, respectively. Whereas, a free triangular mesh with a minimum element size of 90 μm has been applied to layers laid on the outer edge of the diaphragm and swept down to the substrate layer.

A two-step study with parametric sweep has been employed to evaluate the effect of the electrical DC bias to the resonant frequency of the diaphragm. The terminal voltage *V*_b_ of IDT1 shorted with IDT3 has been varied within the range of ±8 V with a step size of 2 V while leaving the terminals of IDT2 and IDT4 electrically floating. 

As a first step, a stationary study has been employed to analyse the mechanical effect of the electric static load, i.e., an electrically induced prestress, on the diaphragm.

**Figure 8:**
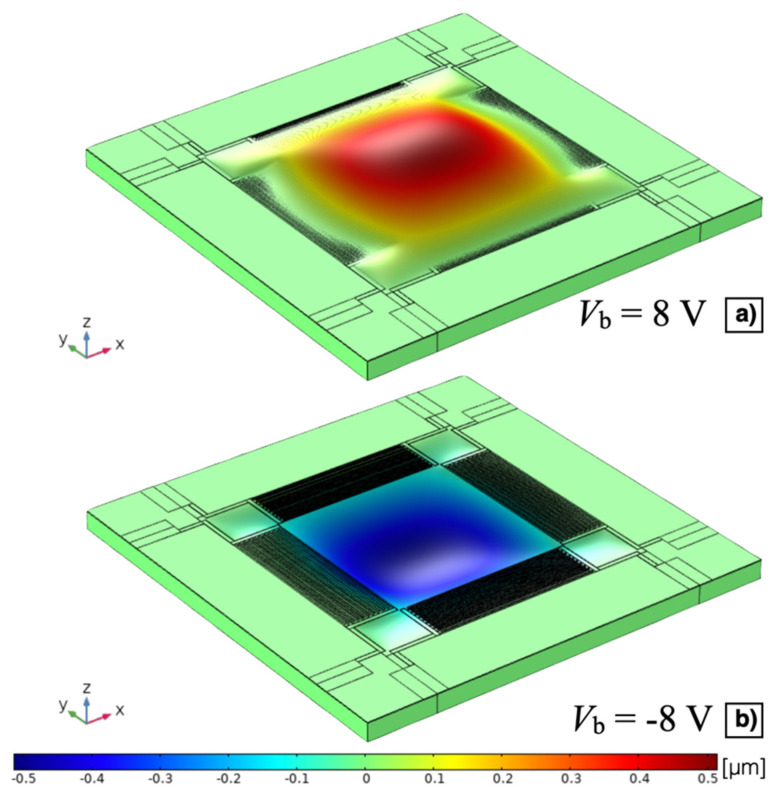
Z-axis displacement, not in true scale, of the MEMS device with *V*_b_ = 8 V (**a**) and *V*_b_ = −8 V (**b**).

**Figure 9:**
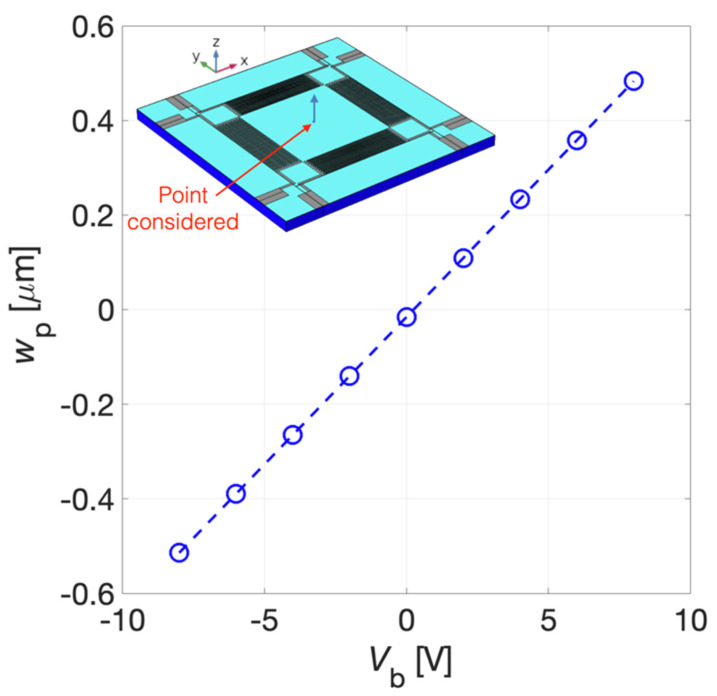
Z-axis displacement *w*_p_ of the point reported in the inset as a function of the bias voltage *V*_b_.

The stationary study results of the z-axis displacement for *V*_b_ = 8 V and *V*_b_ = −8 V have been reported with a 3D representation, not in true scale, in Figure 8a,b, respectively. It can be noticed that, as expected, the convexity of the diaphragm deflection is function of the sign of the applied bias voltage *V*_b_ due to the induced planar static compressive or tensile stress. The z-axis displacement *w*_p_ of the point laid on the top of the AlN surface in the centre of the diaphragm as a function of the bias voltage *V*_b_ is plotted in Figure 9. A displacement of 0.48 μm and −0.51 μm has been obtained at *V*_b_ = 8 V and *V*_b_ = −8 V, respectively.

With *V*_b_ = 0 V, i.e., without electrically induced prestressed, *w*_p_ is equal to −15 nm due to the gravity effect included in the simulation. 

**Figure 10:**
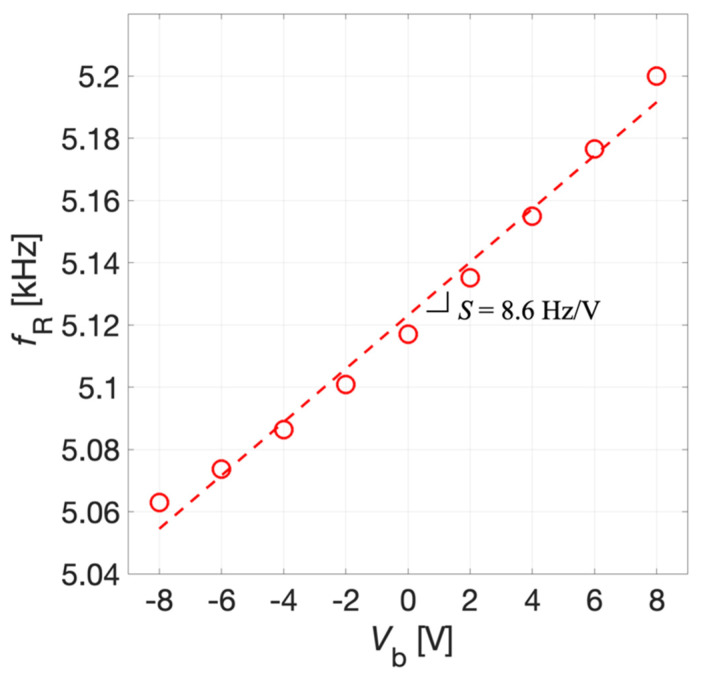
Simulation results of the resonant frequency *f*_R_ of the first eigenmode of the diaphragm as a function of the bias voltage *V*_b_.

As a second step, an eigenfrequency study has been employed to compute the first flexural mode of the structure considering the influence of the electric static load previously evaluated by means of the stationary study. The simulation results of the prestressed eigenfrequency study related to the first eigenmode of the structure are reported in Figure 10.

Specifically, the mechanical resonant frequency *f*_R_ of the piston-like first flexural vibrational mode of the diaphragm is plotted versus *V*_b_. The estimated resonant frequency varies from 5.06 kHz for *V*_b_ = −8 V up to 5.19 kHz for *V*_b_ = 8 V. Therefore, by adjusting the voltage *V*_b_ it is possible to electrically tune the resonant frequency of the diaphragm. As expected, this could provide the system with the capability of reaching the coupling between the series and parallel resonant frequencies of a piezoelectric MEMS acoustic transceiver. The tuning sensitivity *S* = 8.6 Hz/V of the system defined as the linearized ratio between the resonant frequency shift and the applied bias voltage has been estimated by taking the angular coefficient of the linear fitting of simulated data shown in Figure 10.

## 4. Experimental Results

The possibility to improve the receiving-transmitting effectiveness through an applied DC bias voltage *V*_b_ was experimentally investigated by testing the piezoelectric MEMS device in both acoustic receiver and transmitter modes.

**Figure 11:**
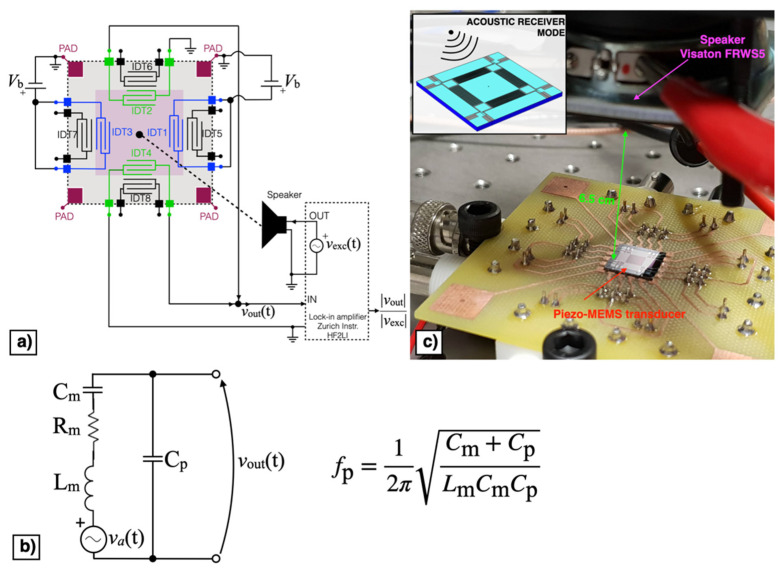
Block diagram (**a**), equivalent Butterworth–Van Dyke (BVD) model (**b**) and experimental set-up (**c**) of the piezoelectric MEMS device in acoustic receiver mode.

The block diagram of the piezoelectric MEMS device configured as acoustic receiver is reported in Figure 11a. In receiver mode the direct piezoelectric effect was exploited by measuring the voltage signal *v*_out_(t) at frequency near the mechanical resonant frequency. The MEMS device can be represented by the equivalent Butterworth–Van Dyke model (BVD) reported in Figure 11b, where the effective mass, mechanical damping, and elastic compliance are represented by the inductance *L*_m_, resistance *R*_m_, and capacitance *C*_m_, respectively. The force induced by the impinging acoustic signal is represented by the voltage *v*_a_(t) in the mechanical branch while the parallel capacitance *C*_p_ represents the dielectric nature of the piezoelectric material. According to such an equivalent circuit, the piezoelectric acoustic device, under voltage readout, displays the highest receiving response at the parallel resonance *f*_p_ [29], defined as:

(1) $$f_{p}=\frac{1}{2\pi }\sqrt{\frac{C_{p}+{\;C}_{m}}{L_{m}C_{m}C_{p}}}$$

A sinusoidal excitation voltage *v*_exc_(t) with peak amplitude *A*_exc_ = 1 V and frequency *f*_exc_ within the bandwidth 5.3–5.6 kHz, provided by the lock-in amplifier (HF2LI, Zurich Instruments: Zurich, Switzerland), was applied to a speaker (FRWS5, Visaton: Haan, Germany) with a flat response in the frequency region of interest placed at 6.5 cm above the diaphragm, as shown in Figure 11c.

**Figure 12:**
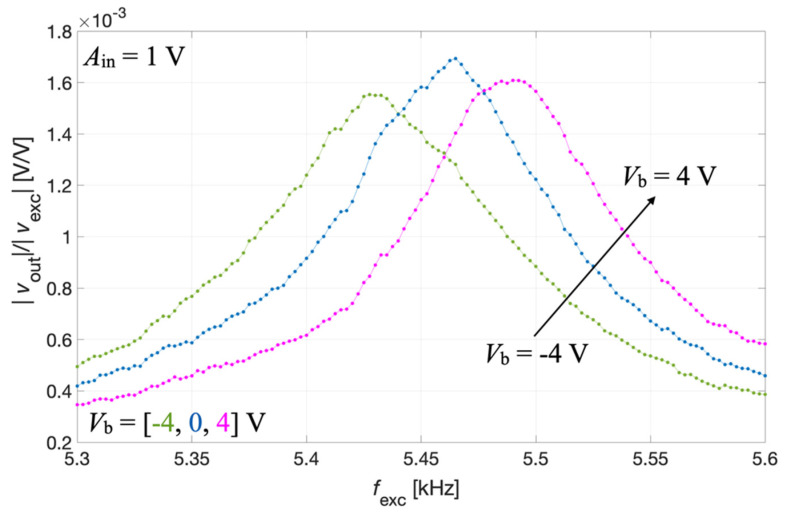
Measured magnitude ratio |*v*_out_|/|*v*_exc_| as a function of the frequency *f*_exc_ for the acoustic receiver mode at different values of the bias voltage *V*_b_.

The output voltage signal *v*_out_(t) was measured across the parallel connection of IDT2 and IDT4, while the bias voltage *V*_b_ was applied between IDT1 shorted with IDT3 and the silicon pad using a power supply (Polytec: Grenoble, France). The acquired voltage *v*_out_(t) was synchronously demodulated with the excitation signal *v*_exc_(t) by the lock-in amplifier, thus providing the magnitude ratio |*v*_out_|/|*v*_exc_| of the resulting receiving transfer function which is plotted as a function of *f*_exc_ for different values of *V*_b_ in Figure 12.

The results of Figure 12 show that by acting on *V*_b_ it is also possible to electrically tune the resonant frequency of the piezoelectric MEMS device configured as an acoustic receiver. 

**Figure 13:**
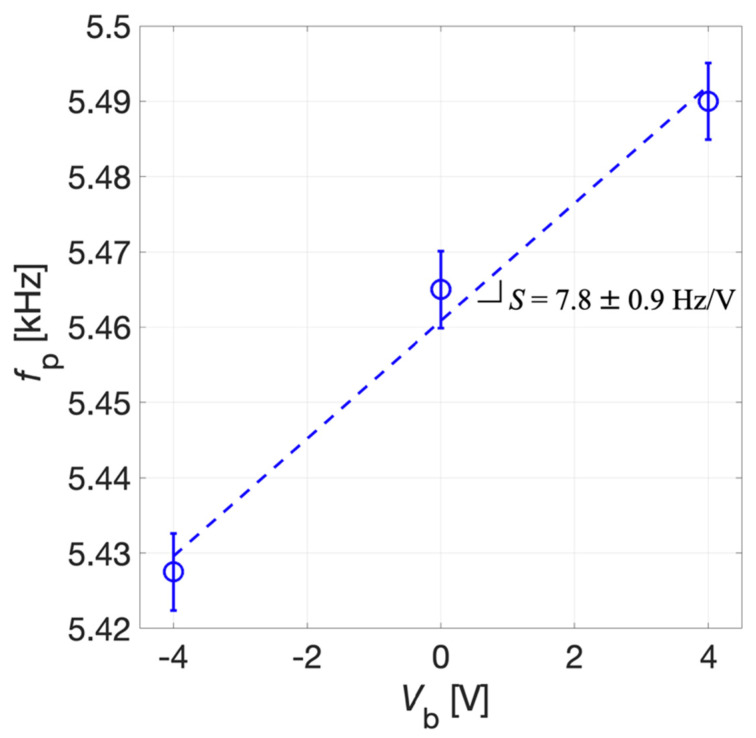
Measured parallel resonant frequency *f*_p_ (circles) and linear fitting (dotted line) as a function of *V*_b_. The error bars extend one standard deviation σ on each side of the experimental data.

The tuning sensitivity *S* was derived by the linear fitting of experimental data reported in Figure 13. The uncertainty for *f*_P_ was estimated as σ = 5 Hz, and the uncertainty of *S* was obtained exploiting the error propagation approach [40]. The tuning sensitivity *S* results 7.8 ± 0.9 Hz/V for the receiver mode. Given the electrical constraints imposed for the FEM simulation reported in Section 3 the simulated mechanical resonant frequency *f*_R_ is expected to approach the parallel resonant frequency *f*_P_ defined in Equation (1). The obtained values of sensitivity show a good agreement between simulated and experimental results, demonstrating that a tunability of the parallel resonant frequency can be obtained in the explored range for *V*_b_. Discrepancies between the simulated and experimental results of *f*_p_ are probably related to the tolerances introduced by the fabrication process of the device which were not taken into full account in the simulations.

**Figure 14:**
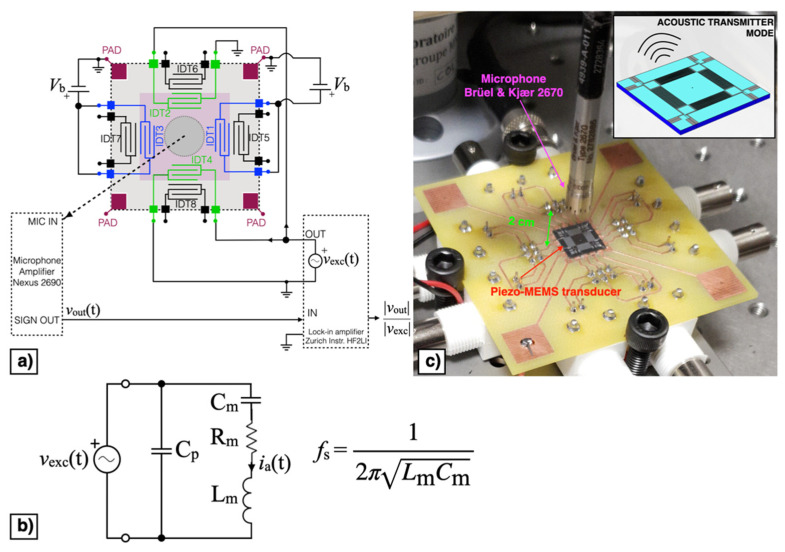
Block diagram (**a**), equivalent BVD model (**b**) and experimental set-up (**c**) of the piezoelectric MEMS device in acoustic transmitter mode.

The block diagram of the piezoelectric MEMS configured as acoustic transmitter is reported in Figure 14a. In transmitter mode the converse piezoelectric effect was exploited by applying the alternating excitation voltage *v*_exc_(t) at frequency near the mechanical resonant frequency. The MEMS device can be represented by the equivalent BVD model of Figure 14b. The velocity of the diaphragm causing the emitted acoustic signal is represented in electrical formalism by the current *i*_a_(t). According to such an equivalent circuit, the piezoelectric acoustic device, under voltage excitation, exhibits the highest transmitting output at the series resonance *f*_s_ [29] defined as:

(2) $$f_{s}=\frac{1}{2\pi }\;\frac{1}{\sqrt{L_{m}C_{m}}}$$

**Figure 15:**
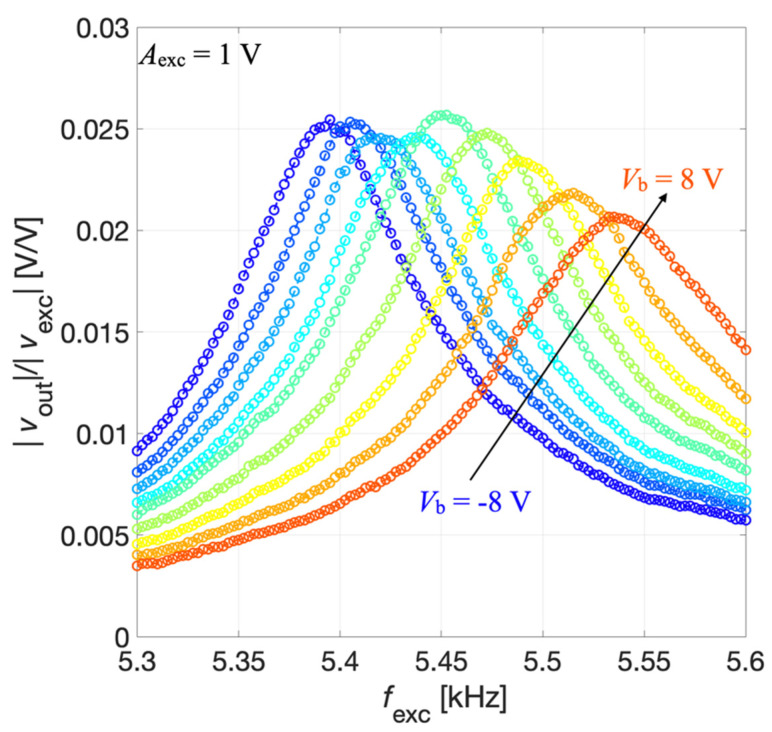
Measured magnitude ratio |*v*_out_|/|*v*_exc_| as a function of *f*_exc_ for the acoustic transmitter mode at different values of *V*_b_.

The excitation voltage *v*_exc_(t) was applied by means of the lock-in amplifier to the parallel connection of IDT2 and IDT4. The DC bias voltage *V*_b_ was applied between IDT1 shorted with IDT3 and the silicon pad and swept within the range of ± 8 V with a step size of 2 V. The generated acoustic signal was measured by a microphone (2670, Brüel & Kjaer: Nærum, Denmark) placed at 2 cm above the diaphragm, as shown in Figure 14c. The microphone output was fed to an amplifier (Nexus 2690, Brüel & Kjaer: Nærum, Denmark) set with a sensitivity of 1 V/Pa. The measured output signal *v*_out_(t) was fed to the lock-in amplifier input for synchronous demodulation with the excitation signal. The magnitude ratio |*v*_out_|/|*v*_exc_| of the resulting transmitting transfer function is reported as a function of *f*_exc_ for different values of *V*_b_ in Figure 15.

The results of Figure 15 show that by acting on the prestress caused by the bias voltage *V*_b_ it is possible to electrically tune the resonant frequency of the piezoelectric MEMS also in transmitter mode. Considering the maximum of the magnitude of the transmitting transfer function *v*_out_/*v*_exc_, the series resonant frequency *f*_s_ was estimated. 

**Figure 16:**
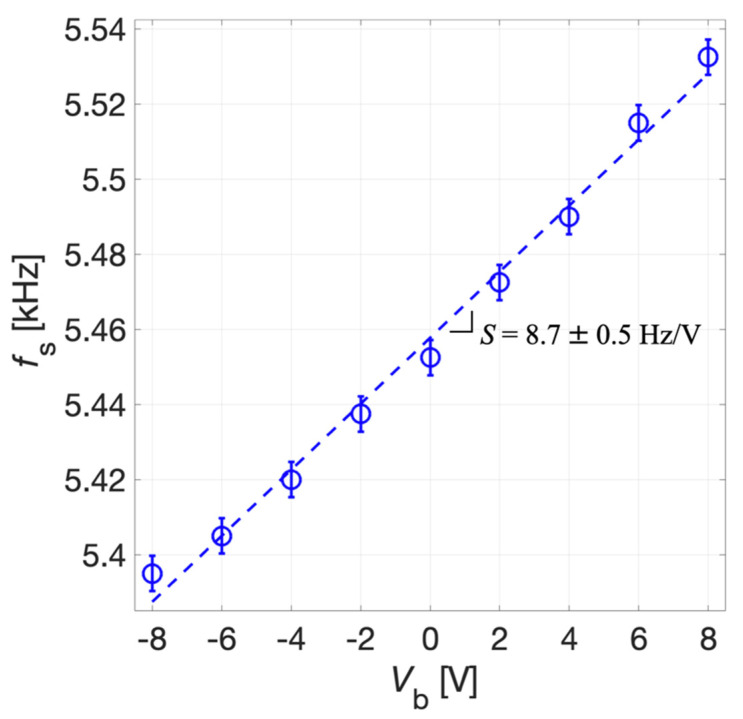
Measured series resonant frequency *f*_s_ (circles) and linear fitting (dotted line) as a function of *V*_b_. The error bars extend one standard deviation σ on each side of the experimental data.

The tuning sensitivity *S* of the system was derived by the linear fitting of experimental data reported in Figure 16. The uncertainty for *f*_S_, as for the receiver mode, was estimated as σ = 5 Hz. The tuning sensitivity *S* results 8.7 ± 0.5 Hz/V for the transmitter mode. The reported data demonstrates that a tunability of about 130 Hz can be obtained in the explored range for *V*_b_. The obtained experimental values of *S* in the receiver and transmitter modes, taking into account their uncertainties, are compatible with each other in metrological sense [40] and closely approach the simulated value.

**Figure 17:**
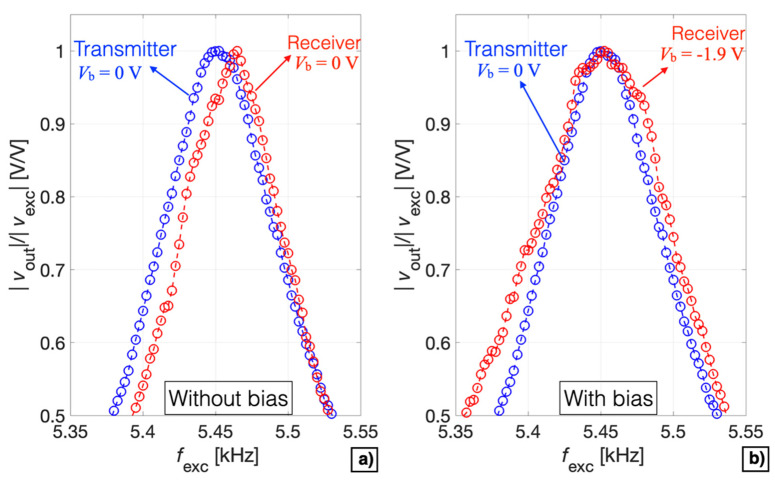
Comparison between the receiver and transmitter mode in terms of the normalized measured magnitude ratio as a function of the frequency *f*_exc_ without (**a**) and with (**b**) the tuning effect induced by *V*_b_.

The measured tuning sensitivities and frequency shifts obtained in both receiver and transmitter modes demonstrate that matching of the series resonant frequency with the parallel resonant frequency can be obtained by acting on the bias voltage in either one of the two working modes. A comparison between the receiver and the transmitter modes in terms of the normalized measured magnitude ratio as a function of the frequency *f*_exc_ without and with the applied tuning by *V*_b_ is reported in Figure 17a,b, respectively.

Specifically, a bias voltage *V*_b_ = −1.9 V was applied to the device configured as receiver to match the resonant frequency of the device configured as transmitter. Therefore, by electrically tuning *V*_b_ it is possible to finely control the resonant frequency of the device, thus obtaining the optimal acoustic emission and detection characteristics with the same operating frequency in both voltage-mode driving and sensing.

## 5. Conclusions

This work has presented a technique to electrically tune the resonant frequency of a piezoelectric MEMS acoustic transducer to obtain matching between the series and parallel resonant frequencies. The piezoelectric MEMS device has been fabricated with the PiezoMUMPs technology exploiting a doped silicon diaphragm with an AlN piezoelectric layer deposited on top. Electrodes disposed symmetrically with respect to the centre of the diaphragm allow for actuating and sensing. By applying a bias voltage *V*_b_ between the bottom doped silicon layer and top electrodes on the AlN layer, an electrically-controllable stress can be induced into the diaphragm, thus leading to the tuning of the resonant frequency.

The working principle of the proposed technique has been studied by 3D finite element modelling in COMSOL Multiphysics^®^ and experimentally verified configuring the piezoelectric acoustic transducer in both receiver and transmitter modes.

Experimental results have shown a tuning sensitivity *S* = 7.8 ± 0.9 Hz/V in receiver mode, whereas a frequency shift of 130 Hz for *V*_b_ = ±8 V and a tuning sensitivity *S* = 8.7 ± 0.5 Hz/V have been reached in transmitter mode. A comparison between the receiver and the transmitter modes has been performed by applying a bias voltage *V*_b_ = −1.9 V to the device configured as receiver to match the resonant frequency of the device configured as transmitter, thus obtaining the optimal acoustic emission and detection characteristics with the same operating frequency in voltage-mode driving and sensing. 

Taking advantage of the non-directional response in the low-frequency range, the proposed device can be employed in pulsed-echo mode as a proximity/presence, or gesture detector. Furthermore, the proposed technique can be transferred to a properly down-scaled structure to obtain a tunable piezoelectric micromachined ultrasound transducer (PMUT).
